# Improving the quality of health information: a qualitative assessment of data management and reporting systems in Botswana

**DOI:** 10.1186/1478-4505-12-7

**Published:** 2014-01-30

**Authors:** Jenny H Ledikwe, Jessica Grignon, Refeletswe Lebelonyane, Steven Ludick, Ellah Matshediso, Baraedi W Sento, Anjali Sharma, Bazghina-werq Semo

**Affiliations:** 1 Department of Global Health, University of Washington, 901 Boren Avenue, Seattle, Washington 98104, USA; 2 Botswana International Training and Education Center for Health (I-TECH), Suite 46 Riverwalk, Private Bag 324, Gaborone, Botswana; 3 Botswana Ministry of Health, Private Bag 0038, Gaborone, Botswana; 4 Botswana Ministry of Local Government, Private Bag 434, Gaborone, Botswana; 5 University of Botswana, Private Bag UB 0022, Gaborone, Botswana

**Keywords:** Data quality, Health information system, Monitoring and evaluation

## Abstract

**Background:**

Ensuring that data collected through national health information systems are of sufficient quality for meaningful interpretation is a challenge in many resource-limited countries. An assessment was conducted to identify strengths and weaknesses of the health data management and reporting systems that capture and transfer routine monitoring and evaluation (M&E) data in Botswana.

**Methods:**

This was a descriptive, qualitative assessment. In-depth interviews were conducted at the national (n = 27), district (n = 31), and facility/community (n = 71) levels to assess i) M&E structures, functions, and capabilities; ii) indicator definitions and reporting guidelines; iii) data collection forms and tools; iv) data management processes; and v) links with the national reporting system. A framework analysis was conducted using ATLAS.ti v6.1.

**Results:**

Health programs generally had standardized data collection and reporting tools and defined personnel for M&E responsibilities at the national and district levels. Best practices unique to individual health programs were identified and included a variety of relatively low-resource initiatives such as attention to staffing patterns, making health data more accessible for evidence-based decision-making, developing a single source of information related to indicator definitions, data collection tools, and management processes, and utilization of supportive supervision visits to districts and facilities. Weakness included limited ownership of M&E-related duties within facilities, a lack of tertiary training programs to build M&E skills, few standard practices related to confidentiality and document storage, limited dissemination of indicator definitions, and limited functionality of electronic data management systems.

**Conclusions:**

Addressing fundamental M&E system issues, further standardization of M&E practices, and increasing health services management responsiveness to time-sensitive information are critical to sustain progress related to health service delivery in Botswana. In addition to high-resource initiatives, such as investments in electronic medical record systems and tertiary training programs, there are a variety of low-resource initiatives, such as regular data quality checks, that can strengthen national health information systems. Applying best practices that are effective within one health program to data management and reporting systems of other programs is a practical approach for strengthening health informatics and improving data quality.

## Background

The World Health Organization has identified health information systems as one of the six key attributes, or building blocks, of a health system [1,2]. The other building blocks are health workforce, leadership and governance, health service delivery, health systems financing, and access to essential medicines. While each of the six building blocks are essential, health information systems are critical for decision-making within each of the other five building blocks, hence forming the foundation of health systems [3]. High quality health information is critical for addressing global health challenges and building strong public health systems [4]. It is necessary for monitoring program goals and objectives, guiding evidence-based program management, and ensuring appropriate policy formulation and resource allocation.

Health information systems rely on multiple sources of data such as household surveys, vital registration, census, and routine monitoring and evaluation (M&E) of health services for information [5]. Routinely collected M&E data can provide important information related to the delivery of national health programs when data are of high quality. Data quality is a complex construct, which encompasses multiple dimensions, including accuracy, reliability, precision, completeness, timeliness, integrity, and confidentiality [6]. Yet, in many resource-limited settings, ensuring data of sufficient quality for meaningful interpretation remains a challenge [5].

There is a growing body of literature investigating the quality of routinely collected M&E data in various countries [7-12]. Many of these have focused on selected dimensions of data quality such as accuracy [7-12], completeness [7,9-11], and timeliness [12]. While documenting and quantifying data quality is important, there is also a need to examine the underlying factors within the health system that influence data quality, in order to establish best practices and implement simple interventions for improving data quality. There are five functional components of data management and reporting that must be established for a health system to produce quality information for decision-making: i) M&E structures, functions, and capabilities; ii) indicator definitions and reporting guidelines; iii) data collection and reporting forms and tools; iv) data management processes; and v) links with the national reporting system [6]. Understanding the strengths and weaknesses of these functional components within health information systems can lead to improved data quality through targeted systems-strengthening activities. The need for structured evaluations focusing on health information systems to determine factors affecting performance and to identify best practices has been noted in the literature [13,14].

To better understand the factors that influence data quality within the routine M&E systems in Botswana, an assessment was conducted of the data management and reporting systems involved in capturing and transferring health data from the point of generation at the health facility or community level to the point of incorporation into national health statistics. The objective was to identify strengths and weakness related to the five functional components of a data management system.

## Methods

This descriptive and qualitative assessment was conducted to provide information on the health data management and reporting systems that capture and transfer routine M&E data in Botswana. The assessment was approved by the Botswana Health Research and Development Committee. It was conducted by the International Training and Education Center for Health in Botswana, a collaboration between the University of Washington and the University of California, San Francisco.

Data were collected through in-depth interviews within three levels of the health system: national, district, and facility/community levels. In total, 129 interviews were conducted; comprising of 27 national-level, 31 district-level, and 71 facility/community-level interviews. Interviews were conducted with public servants; individuals working at private health facilities; and individuals working within civil society at nongovernmental, community-based, and faith-based organizations. The following programmatic areas were included as part of the assessment: antiretroviral (ARV) treatment, tuberculosis (TB), sexually transmitted infections (STI), prevention of mother-to-child transmission(PMTCT) of HIV, HIV testing and counseling (HTC), condom distribution, behavior change and information communication, blood safety, orphans and vulnerable children, and community home-based care. The *Routine Data Quality Audit Tool* was used to guide interviews [6]. Information was collected on five areas of data management and reporting systems: i) M&E structures, functions, and capabilities; ii) indicator definitions and reporting guidelines; iii) data collection and reporting forms and tools; iv) data management processes; and v) links with the national reporting system [6]. Table 1 presents an overview of the questions addressed within each of these five functional components. Each interview was conducted face-to-face by a trained interviewer, in the presence of a note taker. With permission from each interviewee, the interview was recorded and transcribed. All interviewees were asked to provide recommendations for other individuals to be included in the assessment.

**Table 1:** Questions addressed within each of these five functional components of a data management system needed to ensure data quality^*^

**Five functional components of a data management and reporting system**	**Questions**
M&E structures, functions, and capabilities	• Are key M&E and data management staff identified with clearly assigned responsibilities?
	• Have the majority of key M&E and data management staff received the required training?
Indicator definitions and reporting guidelines	• Are the operational indicator definitions meeting relevant standards that are systematically followed by all service points?
	• Has the program/project clearly documented (in writing) what is reported to who, and how and when reporting is required?
Data collection and reporting forms and tools	• Are there standard data-collection and reporting forms that are systematically used?
	• Is data recorded with sufficient precision/detail to measure relevant indicators?
	• Are data maintained in accordance with international or national confidentiality guidelines?
	• Are source documents kept and made available in accordance with a written policy?
Data management processes	• Does clear documentation of collection, aggregation, and manipulation steps exist?
	• Are data quality challenges identified and are mechanisms in place for addressing them?
	• Are there clearly defined and followed procedures to identify and reconcile discrepancies in reports?
	• Are there clearly defined and followed procedures to periodically verify source data?
Links with the national reporting system	• Does the data collection and reporting system of the program/project link with the National reporting system?

^*^Based on the *Data Quality Audit Tool*[6].

For the 27 national-level interviews, 17 (63%) were with organizations in the public sector and 10 (37%) were with civil society organizations. Purposive sampling was used to identify the key person or persons at the national level involved in monitoring and evaluating the ten health programs included in the assessment.

For district-level interviews, a random sampling approach was used to select three districts (Francistown, Kanye, and Chobe) of the 20 main health districts and one of the eight sub-districts in the country (Moshupa). Of the 31 district-level interviews conducted, 24 (77%) were with organizations in the public sector and 7 (23%) were with civil society organizations. Interviewees included leadership at the district health management team (DHMT) and other individuals with data-related responsibilities, such as district M&E officers and programmatic focal persons.

Within each of the three districts selected for the district-level assessment, facility/community level interviews were conducted within at least one public hospital, two public clinics, one health post, and one mobile clinic. The public clinics were randomly selected. Health posts (a health care facility providing a minimum package of services) and mobile clinics were purposively selected as identified by the leadership of the DHMT in each of the districts. Additionally, in each district, convenience sampling was used to select one private sector health facility providing ARV services on behalf of the Government of Botswana as part of a public-private partnership initiative. Of the 71 facility/community-level interviews, 48 (68%) were in the public sector, 5 (7%) were in the private sector, and 18 (25%) were within civil society organizations.

Over 300 reference materials, including registers, tools, and electronic systems, were collected during the interviews. These reference materials, along with written M&E policies and procedures, historical reports, and written feedback were reviewed to triangulate and contextualize findings from the interviews.

ATLAS.ti (Version 6.1, Scientific Software Development) was used to assist with systematic analysis of the qualitative data. A framework analysis was conducted to answer key questions related to the five functional components of data management and reporting systems. Framework analysis links data to the source, enabling the visual presentation of data specific to the different health system levels (national, district, and facility/community level) and by sector (private, public, and non-governmental) per health program [15,16]. After reviewing the data, a standardized code list was developed to identify recurrent and important themes. Transcripts were reviewed to identify, code, and index responses. Brief summaries of the data were generated and synthesized using a framework focused on generating policy and practice-oriented findings.

## Results

### M & E structures, functions, and capabilities

Key M&E and data management responsibilities at the national level were defined; however, there were limited human resources available for these activities and the majority of positions were donor-funded, fixed-term appointments. At the district level, responsibilities were also defined but varied substantially by district and health program. For example, the district M&E officer may be responsible for receiving programmatic data in one district, while the same type of data would be received by the community health nurse or programmatic focal person in another district. At both the national and district level, staff with M&E and data management responsibilities often reported having competing priorities, which interfered with routine M&E functions. At the facility/community level, M&E and data management responsibilities were generally not clearly assigned, and there was often a lack of ownership of M&E-related tasks. Health workers did not necessarily view the recording of patient and health facility information in registers as one of their job responsibilities. It was reported that this information is often entered long after a client receives services, particularly if there are long queues at a facility. As three district-level interviewees remarked:

“Really poor documentation from the facilities is what makes data to be of very low standard”.

“Nurses are saying that data is not part of their job description, so they don’t really know why they should keep recording it because it takes time”.

*“When you look at the National PMTCT uptake, you look at* [District x]*, they always have a low performance. It isn’t that they are not doing the job, they just don’t document. They don’t record”.*

Information technology (IT) support was found to be insufficient at all levels, which was a challenge for maintaining and updating health-related electronic systems. Respondents from facility to national level reported data loss due to computer crashes, viruses, and misfiled electronic data. Without IT support to maintain computer systems and functionality, health worker access to computer-based files was limited for various periods of time.

The training and skill level of key M & E and data management personnel varied substantially. Due to a lack of M&E courses and training programs at tertiary education institutions in Botswana, M&E-related training was generally through workshops and short courses. Most M&E staff at the national and district level had received basic training in M&E, but their analytic skills were reported to be notably weak. There was an acknowledgment of the need to train individuals at the facility level, particularly on the data collection tools. However, it was noted that the transfer of health workers between districts and facilities often led to gaps in data recording and reporting as trained and experienced health workers transferred out of facilities.

One approach identified by interviewees at the facility to address challenges related to having trained staff to carry out M&E responsibilities was attention to staffing patterns. At one facility, having a posted schedule indicating who was responsible for compiling the monthly PMTCT statistics was reported to help ensure that M&E related tasks were carried out appropriately. Having an adequate number of staff to cover all responsibilities, including M&E, was reported to improve data quality. For example, one community health nurse at the district-level stated:

“*Timeliness, I had that issue, but it is sorted out since two months ago because now we have many nurses. Really, it* [timeliness] *was being affected by the fact that there was a shortage* [of nurses] *at the clinics”.*

An alternative approach highlighted by ARV program staff for capturing data at the facility level was to have data entry clerks instead of clinical staff assisting with data capture.

Another best practice was building recognition for the importance and uses of health data for evidence-based decision-making. Several national programs reported that they conducted M&E-specific workshops for district-level staff. For example, the national STI unit holds an annual M&E workshop in which district staff present key STI data from their districts and discuss their successes, challenges, and future plans for improving the quality of STI care. Similar quarterly meetings were reported by the national TB program. There were also reports of workshops for staff at the facilities to highlight the importance of M&E and to provide training on data collection tools. One national-level interviewee described a training for the data entry clerks supporting the ARV program in which the importance of capturing accurate CD4 data was exemplified:

*“We illustrated the importance of the baseline CD4 data and the impact that has on the whole ministry, how in a survival analysis that we did we could prove that patients have a high risk of death if they come in with a lower CD4 count, and if they come in earlier* [with a higher CD4 count] *they have a much longer life expectancy”.*

However, the interview highlighted that cost was a limiting factor for these national-level training initiatives and emphasized the importance of decentralizing leadership of these activities to staff at the district level as a less resource-intensive approach.

### Indicator definitions and reporting guidelines

When asked about the existence and availability of indicator definitions for key variables, responses were mixed, with some participants indicating that these are available and widely shared, while others reported not having these definitions. Examples of the divergent responses are presented below:

“Yes, in the guidelines there are definitions…when we audit, we go around discussing the variables so that the report should come at a quality standard and so that we understand the variables in one way, knowing that we are all doing the same thing”.

*“No. Just last week I was asking how I am supposed to evaluate programs when I don’t have their indicators. The only program that I have indicators of so far is* [Program X]*. I have those that I need for* [Program X] *to show where we are and how effective it is”.*

Interviewees at the national level were more likely to respond that indicator definitions were available than those at the district and facility/community levels. In some cases, well-developed indicators had been developed at the national level but not well disseminated.

Reporting guidelines appeared to be widely known and nationally consistent. In some cases, this information had been conveyed in writing, but more often it was common knowledge. As indicated by a district-level officer:

*“Even though they were not written, they* [facility level staff] *know that by the 5th of every month, a complete monthly report is supposed to be here. Not submitted in parts; but the report should be complete. So they know that from the 1st to the 5th they are supposed to submit the reports”.*

However, knowledge of reporting deadlines did not seem sufficient to encourage timely reporting, with late reporting appearing to be common at all levels and across all programs within the health system. Few repercussions were acknowledged for dealing with late reporting. When one member of the DHMT was asked if there are any measures in place to deter late reporting, the interviewee replied: *“Aaah! There is nothing at all”.*

A best practice was the integration of indicator definitions into a general guide, which created a single source of information related to the program. For example, the national TB program manual and the national HTC guidelines both include not only a thorough discussion of programmatic implementation, but also indicator definitions. The adoption of indicators that correspond to international indicators, such as those established related to blood safety, were also identified as a best practice. Additionally, some programs have reporting requirements listed on the reporting forms to provide clarity for health workers.

### Data collection and reporting forms and tools

Standardized data collection and reporting tools were available for most programs and were, generally, reported to be used by health workers. When these did not exist or were out-of-stock, health workers would, however, commonly collect data in notebooks. It was reported that some health workers preferred using notebooks as compared to the standard tools, particularly in cases in which they did not believe the data collection tools were sufficient for capturing necessary information. As stated by one district-level interviewee:

“Sometimes you’ll find that the national registers are blank, but they’ve opened little hardcover books that they keep the information in and then they don’t transfer it to the national registers”.

While standard tools were generally available, staff at the district and facility level reported that the tools frequently change. Ensuring that the most up-to-date version was being used was reported to be a challenge.

Some programs did report that the data collected lacked sufficient precision for meaningful interpretation. For example, interviewees reported that the STI data was insufficient for tracking STI trends given that many conditions are simply collapsed together and reported as “other STIs”. As stated by one interviewee*:*

*“Some of the conditions are lumped together, so now that when you analyze the data, it will just say ‘other STIs,’… Now if you want to…maybe take out to* [select] *genital warts, it cannot give you any information because it has been lumped together”.*

There were no standard practices identified to ensure that data were maintained in accordance with any confidentiality guidelines nor were there standard practices for maintaining and storing source documents. There was no reported government policy on how long data should be stored or how often it should be backed up to protect against data loss. For those who indicated that they were routinely backing up their data, a variety of methods were reported, including compact discs and thumb drives/memory sticks. As one participant indicated:

*“As soon as I finish with a report I put it in a* [memory] *stick”.*

While memory sticks were commonly reported as a method for backing up information, there was acknowledgment that they are often low quality, have limited storage space, are susceptible to viruses, and are easy to lose. As described by another participant:

*“I was just saving it* [data] *on a stick, but it is lost now”.*

Best practices related to the forms and tools included conducting a consultative process with staff at the district and facility level when these items are being revised to allow for input from those who use the forms and tools. Additional best practices used by some programs included having a unique number for each form and register, along with an accurate document version number to help alleviate confusion. Interviewees reported some best practices as ensuring that tools include key definitions, guidelines for collating data, and steps to be taken when additional forms are needed. Nevertheless, interviewees did report that when this information was on the back of the forms, it was often ‘lost’ when forms were photocopied. Additionally, several programs such as the TB and HTC programs, included M&E sections in their national program guidelines that provide information on data collection tools.

### Data management processes

Some programs provided documentation of steps in the data management process, but these generally did not include clear guidance on procedures for collection, aggregation, and manipulation of the data. For example, there was little evidence of guidelines for cleaning, editing, and documenting changes on source documents, raw data, and reports. There were also no clearly identified mechanisms for addressing data quality challenges, such as data completeness or double counting. Data completeness was highlighted as a challenge. Since many facilities report late, districts often submit reports to the national level that only contain data for a portion of facilities that should be included. The following responses from interviewees demonstrate issues of data completeness:

“This month a facility reports, next time it doesn’t report. As we are compiling, it’s not like all of the facilities reported”.

“*There is no written policy on what you are supposed to do when the report is late or when you discover some discrepancies”.*

“I sometimes have to use the previous month’s data so that I also meet the deadline”.

The reports sent to the national level, did not commonly contain information on which facilities reported and there was no standard way of tracking or reporting completeness in terms of coverage of the facilities. At district and national level, some interviewees reported double-checking the data that they enter into the computer for accuracy. There did not appear to be clearly defined and documented procedures to periodically verify source data. At the national level, some guidance on data audit and review of source documents is provided. However, there is no regular schedule for these audits.

### Links with the national reporting system

Vast amounts of data are being generated at the facility/community level and transferred to the district and then national level. For example, the Out-Patient and Preventative Health Statistics Monthly Summary Form, which is completed by all government health facilities (over 600 facilities), contains almost 300 data elements. Additionally, most health programs also have monthly reporting forms that are program-specific. It was not unusual for a single data element to be reported on multiple data collection forms. Staff of the district and facility/community level reported receiving only limited feedback, with most feedback being negative and related to the timeliness of data or data errors. There is often no contact made to communicate that a report has been received and reviewed. As one participant stated:

“*If they don’t call me I know I have done a good work, if they call me then I panic; but besides that, I don’t get any feedback”.*

To help manage the large amounts of data being generated and processed through the health information system, some programs have implemented the use of electronic patient-level reporting systems. Multiple systems exist; as stated by one participant:

“Donors are giving us a lot of money for programs and that is why we have had a mushrooming of different databases when we should have a single, standard database”.

These systems are often not integrated across health programs or linked across health facilities, limiting the delivery of coordinated health services. Due to the unreliability of the electronic systems, they often increase the workload of health care workers. They do not eliminate the need for paper-based forms, but rather exist in parallel due to unreliable internet services, insufficient IT support, and lacking computer skills among health care workers. There were reports of data losses from the electronic systems due to malfunctioning as well as backlogs of data not being entered.

It was found that data from services provided in private health facilities, prisons, and military establishments were not well captured. Services provided through civil society organizations were also often not well integrated into the national reporting system. For example, the data flow pathways for these organizations were not standardized and varied by organization. In some cases, the same data elements were reported multiple times at different levels within the national reporting system, which led to a risk of double counting. In other cases, data from civil society organizations were not captured into the national reporting system. Reporting tools also lacked standardization, as one district M&E officer reported:

“We say just bring us the report that you gave to your headquarters and we’ll take what we want from it”.

Given the frequent reliance of some civil society organizations on volunteers, M&E-related capacity was often limited.

A best practice was the utilization of supportive supervision visits to districts and facilities. As a less resource-intensive method of providing feedback, the national ARV program reported providing a monthly telephone call to district focal persons. As stated by one individual:

“If you see that an area has a lot of loss to follow up or a long waiting list…we want to go and find out what is happening and try to help, but we also communicate via the phone. Some we are able to rectify via the phone, some we need to go there and really help them”.

The ARV program also reported disseminating program performance statistics on a monthly basis to a broad list of stakeholders.

## Discussion

Improving data capture, transfer, and feedback systems will strengthen the delivery of health programs by improving the quality of information used to plan national, district, and facility-level programs. This qualitative assessment has demonstrated that while there are a variety of resource-intensive initiatives that can be taken to strengthen national health information systems, there are also many initiatives that are less resource-intensive that can positively affect these systems. This assessment also identified multiple best practices existing within distinct health programs. Applying best practices that are effective within one health program to the data management and reporting systems of other health programs is practical, feasible, and effective in strengthening health information systems.

In Botswana, as in many other countries, human resources are a considerable challenge for maintaining data quality within health information systems [9,11,17,18]. A workforce already burdened by HIV/AIDS-related service provision requires approaches that minimize and streamline data-related tasks. While increasing human resources to support data-related duties may be resource intensive, this assessment highlights the importance of attention to staffing patterns of existing staff, which is a less costly approach. The development of a skills and tasks inventory would be a relatively low-resource first step in clarifying staffing needs for the generation and use of strategic information. Task-shifting has also been shown to be a cost effective strategy for distributing duties within the health sector [19,20]. Data from the present assessment suggest that task shifting of M&E duties at the facility level to paraprofessional cadres, such as data entry clerks, may be an effective strategy to strengthen data quality. This is supported by another study in Botswana, which indicated that lay counselors did a better job with documentation for program monitoring and evaluation than other cadres of health workers [21], as well as data from Malawi which found having data entry clerks improved data quality at ARV clinics [9].

In addition to examining staffing patterns to streamline data-related tasks, the data from this assessment suggests several simple, practical capacity development approaches to strengthen health management systems by improving data demand, use, and quality assurance. This would include decentralization of training and mentoring initiatives from the national to the district level for health workers delivering services at health facilities, which would allow for support at the point of data generation. On-the-job training and mentoring has been shown to be an effective approach for strengthening M&E capacity and ensuring data quality within a national health system [22]. The need for decentralization of capacity building activities has also been supported by data from Malawi [23]. Provision of support at facilities by district level staff would be facilitated through the creation of standardized materials for training and mentoring on health information as well as job aids. Examples of such materials include a single source of information containing indicator definitions, guidelines on collating/aggregating, auditing procedures, as well as other steps of data collection, handling, analysis, and reporting. The development of job aids is supported by a study in Tanzania, the use of standardized analysis templates based on the Millennium Development Goals and local strategic plans contributed to improved data quality [24]. Materials to facilitate data use are particularly warranted given that this assessment, as well as work elsewhere [23], found that ownership of data-related tasks at service delivery sites was a key impediment to data quality. Data use has been shown to be important in facilitating improvements in data quality [18,24].

Resource intensive investments that can improve data quality include strengthening electronic health information systems, investing in tertiary education programs, and harmonizing data collection systems. Computerized point-of-care health information systems, particularly web-based systems, have the potential to dramatically reduce the data collection burden by automating data aggregation and reporting [25]. These systems would also allow for real-time access to data [25,26]. Data from the present assessment indicate that this potential has not yet been reached as the electronic systems in place are incomplete, lack integration, are unreliable, and create a double reporting burden as data are often captured in both paper-based and electronic systems. While advances in information technology can enable large volumes of data to be processed and analyzed quickly, the success is highly dependent on having adequate hardware, sufficient internet access, and common data architecture between systems, IT professionals, and support to ensure systems maintain functionality. These critical success factors remain a challenge in many resource-limited settings including Botswana [27,28]. Mobile phone networks are being successfully used as part of national data collection systems; however, they require substantive investment and maintenance [29]. A simple and regular system of data quality checks may be more cost-effective and reliable in the current context. The lack of training programs related to M&E at the tertiary level was identified in the current assessment as a challenge for ensuring that the health workforce has a good foundation in M&E, which was also highlighted as a challenge for implementing of a comprehensive M&E system in the Botswana 2012 Global AIDS Response Report [28]. Tertiary education programs are costly to develop and the return on the investment is only realized after many years due to the time required for course development and for students to matriculate and move into the job market.

An additional, relatively high-resource investment suggested by the data in the current assessment is the harmonization of data collection systems across health programs. This would include the development of essential data sets, which needs to be a timely, intensive, and consultative process for it to be effective [30]. Data from South Africa have shown that health workers often perceive data collection and collation to have a high work burden [31]. The present assessment suggest that data-related activities are often sacrificed because of the high-time commitment as well as competing priorities. While there is an unquestionable need for data within all levels of a health system, the extensive use of multiple vertical reporting systems puts a burden on the M&E system. The lack of integration of data across different disease program areas has been a common challenge facing national M&E systems [25,31,32]. Therefore, the parallel data systems identified in this assessment increase reporting burden and risk of confusion among facility-level staff [26].

A limitation of this assessment was the focus on programs related to HIV/AIDS only. Nevertheless, it is likely that the findings would be applicable to other health programs. As participants were fully aware of the purpose of the assessment, they may have exhibited a social desirability bias, expressing to interviewers what they know are the policies concerning data management and reporting, rather than explaining the current procedures at their site. Another limitation was that assessment did not include objective measures of data quality. Future data verification exercises are warranted.

This assessment has identified both high and low resource approaches to strengthen health information systems, which will likely need to be addressed in tandem to effect the most substantive and sustainable impact. Data quality is critical in ensuring that appropriate conclusions are drawn from the information captured at the health facility/community and integrated into national reports. Approaches to improve data quality need to be based on scalable solutions which can be effectively used at all levels of the health system [33]. Applying best practices that are effective within one health program to other programs will provide a vehicle for scaling up initiatives. This would include ensuring that staffing patterns are established to meet health information needs, providing need-based supportive supervision along with appropriate IT support, and fostering health data use for evidence-based decision-making. Institutionalizing the best practice of having a single source of information related to indicator definitions, data collection tools, and management processes allows for the use of a standardized data system across health programs. Aligning indicators to international indicators, when possible, will facilitate removal of duplicative collection processes. Finally, inclusion of M&E sections in national program guidelines that provide information on data collection tools, giving forms and registers unique numbers as well as accurate version numbers, along with clear guidance on steps to be taken when additional forms are needed, will help staff to remain informed about data collection guidelines and requirements. Approaches to manage health information systems need to be dynamic and responsive to local data and information needs thorough consultative processes when revising policies, procedures, forms, and tools.

## Conclusions

While there are a variety of resource-intensive initiatives that can be taken to strengthen national health information systems, such as investments in electronic medical record systems and tertiary training programs, there are also many lower cost initiatives that can have substantial, positive impacts on data management and quality. These include implementing staffing patterns that enable adequate human resources for data-related tasks, decentralizing health worker training and mentoring on data quality to the facility and district levels, and developing a comprehensive, single source of information to guide data management. Applying strategies that are effective within one health program to other programs is a practical, feasible, and effective strategy for strengthening health information systems.

## Abbreviations

ARV: Antiretroviral; DHMT: District health management team; HTC: HIV testing and counseling; IT: Information technology; M&E: Monitoring and evaluation; PMTCT: Prevention of mother-to-child transmission; STI: Sexually transmitted infections; TB: Tuberculosis.

## Competing interests

The authors declare that they have no competing interests.

## Authors’ contributions

JHL, BWS, AS and BS, participated in study design, data collection, data analysis, and manuscript writing. JG, RL, SL, EM participated in study development and the revision of the manuscript. All authors read and approved the final manuscript.
